# Histological characteristics of the largest and secondary tumors in radical prostatectomy specimens and implications for focal therapy

**DOI:** 10.1186/s13000-019-0782-8

**Published:** 2019-01-12

**Authors:** Young Hyo Choi, Ji Woong Yu, Byong Chang Jeong, Seong Il Seo, Seong Soo Jeon, Hyun Moo Lee, Hwang Gyun Jeon

**Affiliations:** Department of Urology, Samsung Medical Center, Sungkyunkwan University School of Medicine, 81 Irwon-ro, Gangnam-gu, Seoul, 06351 South Korea

**Keywords:** Focal therapy, Hemiablation, Histological characteristics, Prostate cancer, Radical prostatectomy specimen

## Abstract

**Background:**

Pathological features of prostate cancer in Korean men were analyzed to determine whether identification of tumor volume, Gleason score (GS), focality, and location using radical prostatectomy (RP) specimens can provide useful information for the application of focal therapy (hemiablation).

**Methods:**

From January 2016 to December 2017, 913 patients who underwent RP at a single center were selected for analysis. Patients with prostate-specific antigen levels > 15 ng/mL or those who had received hormone therapy prior to surgery were excluded. Preoperative data and the number, volume, location, and GS of each tumor were recorded.

**Results:**

Overall, 762 RP specimens were examined, and 1448 tumors were identified. The majority of the cases were multifocal (60.5%) and bilateral (82%) in nature. Among the 686 secondary tumors, 250 (36.4%) had a GS ≥7 and 122 (17.8%) had a tumor volume ≥ 0.5 mL. Among the 435 bilateral multifocal cases, secondary tumors on the lobes contralateral to the largest tumor were significant by volume (≥0.5 mL) in 91 (20.9%) cases and by grade (GS ≥7) in 179 (41.1%) cases. There were 102 (23.4%) tumors with a small tumor volume (< 0.5 mL) and Gleason pattern 4 on the lobe contralateral to the largest tumor.

**Conclusions:**

Bilateral and multifocal tumors are a common feature and secondary tumors frequently exhibit clinically significant prostate cancer on RP specimens in Korea. In many cases, secondary tumors on the lobe contralateral to the largest tumor had a high GS and small tumor volume.

## Background

Radical prostatectomy (RP) and whole-gland radiotherapy are currently the standard of care for definitive management of clinically localized prostate cancer. However, whole-gland therapy is often considered an unnecessary treatment or overtreatment for many patients [1]. In the last decade, focal therapy has been evaluated as an alternative treatment for selected men harboring localized prostate cancer. The aim of this tissue-preserving treatment is to maintain effective oncologic outcomes by selectively ablating known disease, while optimizing genito-urinary function [2, 3]. Although partial resection or focal ablation in many solid cancers is acceptable in eligible patients, theoretical objections to focal therapy in prostate cancer include the multifocal nature of the malignancy [4, 5].

The rationale for focal therapy relies on evidence-based elements. First, the natural history of prostate cancer appears to be linked to the index tumor in the majority of men, and secondary low-grade tumors appear to exhibit indolent behavior in most cases [6–8]. Small, low-grade secondary tumors may therefore be clinically irrelevant if the index tumor can be ablated and controlled [9]. Second, there is growing evidence that multiparametric magnetic resonance imaging (mpMRI) detects both high-grade and larger tumors accurately [10, 11]. Detection rates of clinically significant prostate cancer using mpMRI ranged from 44 to 87% in biopsy-naïve men and men with a prior negative biopsy. The negative predictive value of mpMRI for exclusion of significant cancer ranges from 63 to 98% [12].

Although mpMRI is promising for detection of larger tumors, it lacks sufficient sensitivity to detect smaller tumors. Approximately 20% of men had non-index tumors with a Gleason score (GS) ≥7, but detection of these tumors was poor [13]. In addition, Korean patients with prostate cancer tend to have worse disease characteristics than Western men [14, 15]. Korean men appear to have more high-grade tumors and more advanced cases than Western men, after adjusting for various confounding variables, such as age at surgery, preoperative levels of prostate-specific antigen (PSA), and clinical stage [15].

Thus, the aim of this study was to examine the largest tumors and smaller high-grade tumors that are easy to miss with mpMRI through final pathologic assessments of Korean patients who underwent RP. These data may be useful for informing patient selection for focal therapy. For example, data on the number of clinically significant small tumors remaining in untreated lobes following hemiablation would help determine appropriate courses of treatment.

## Methods

### Patients

A total of 913 consecutive patients who underwent RP at a single center between January 2016 and December 2017 were selected for analysis. Thirty-four patients who received hormone therapy before the surgery, one patient with rhabdomyosarcoma, one with no malignancies, and 115 with a PSA level > 15 ng/mL were excluded. The remaining 762 patients were included in the final analysis.

### Data collection

The preoperative clinical features of patients, including age and PSA level, were reviewed from electronic medical records. From the RP specimens, the total number and volume of tumor(s), the highest GS, and the pathological stage were recorded. The GS and volume of each tumor focus were also recorded. Significant tumors were defined as those with either a tumor volume ≥ 0.5 mL or a GS ≥7 (3 + 4) [16].

### Histopathological evaluation

Each prostate was indicated with India ink and fixed in 10% buffered neutral formalin for 24 h. After fixation, the apex and base were amputated and serially sectioned parallel to the urethra. Paraffin-embedded RP specimens were sectioned at 5 μm thickness and stained with hematoxylin-eosin. Seminal vesicles were sectioned parallel to their junction with the prostate and submitted in entirety for evaluation. The remaining specimen was serially sectioned perpendicular to the longitudinal plane of the gland at 3- to 4-mm intervals from the apex to the base. One experienced genitourinary pathologist examined all of the specimens.

The volume of each tumor was calculated using the ellipsoid estimation described by Perera et al. [= k (π/6) × length × width × thickness] [17]. Length was the longest linear dimension of the largest cross-sectional profile for that focus, and width was the second longest linear dimension in the maximum cross-sectional profile, approximately at right angles to the length. Height was defined as the number of cross-sections occupied by each focus multiplied by 0.5 cm, which was the thickness of each tissue block.

When tumor areas were separated by > 4 mm within the same slide or 5 mm in adjacent slides, the areas were regarded as separate tumor foci [18–20]. When multifocal tumors were observed, the primary tumor was defined as the largest tumor as measured by volume, without considering its GS. As secondary tumor was defined as a tumor smaller than the primary tumor. Tumor laterality was divided along the sagittal plane with the urethra as a midline. “Bilateral unifocal” denotes the presence of one tumor crossing the midline and “bilateral multifocal” refers to the presence of at least one tumor in each of the right and left lobes.

### Statistical analysis

The Mann–Whitney U test was used to compare age, PSA, and prostate volume of the different groups. Pearson’s chi-squared test was used to compare characteristics between the two groups based on noncontinuous variables (such as GS and pathological stage). Pearson correlation and linear regression were used to describe the relationship between total tumor volume and index tumor volume. All statistical analyses were performed using IBM SPSS version 20.0 (IBM Corp., Armonk, NY, USA). Two-sided *p* values < 0.05 were considered statistically significant.

## Results

### Baseline characteristics

Baseline characteristics of the 762 RP specimens are presented in Table 1. The median age of the patients was 66 years (range: 43–81 years) and the median PSA was 5.09 ng/mL (range: 0.34–14.95 ng/mL). The mean overall tumor volume was 3.24 mL (standard deviation: 4.16). The majority of the cases were multifocal (60.5%), well-to-moderately differentiated (GS 6 and 3 + 4, 68.3%), and organ-confined (pT2, 73.4%).

**Table 1:** Baseline characteristics of 762 radical prostatectomy specimens

Age, years	
Mean ± SD	65.5 ± 6.7
Median (range)	66.0 (43.0–81.0)
PSA, ng/mL	
Mean ± SD	5.93 ± 3.07
Median (range)	5.09 (0.34–14.95)
Tumor focality	
Unifocal	301 (39.5%)
Multifocal	461 (60.5%)
Tumor laterality	
Unilateral unifocal	111 (14.6%)
Unilateral multifocal	26 (3.4%)
Bilateral unifocal	190 (24.9%)
Bilateral multifocal	435 (57.1%)
Overall tumor volume, ml	
Mean ± SD	3.24 ± 4.16
Median (range)	1.97 (0.01–57.41)
Gleason score	
6 (3 + 3)	86 (11.3%)
7 (3 + 4)	434 (57.0%)
7 (4 + 3)	140 (18.4%)
8	35 (4.6%)
9	67 (8.8%)
Pathologic stage	
pT2a-b	102 (13.4%)
pT2c	457 (60.0%)
pT3a	133 (17.5%)
pT3b	69 (9.1%)
pT4	1 (0.1%)

*PSA* = prostate-specific antigen, *SD* = standard deviation

### Characteristics of individual tumors

**Fig. 1:**
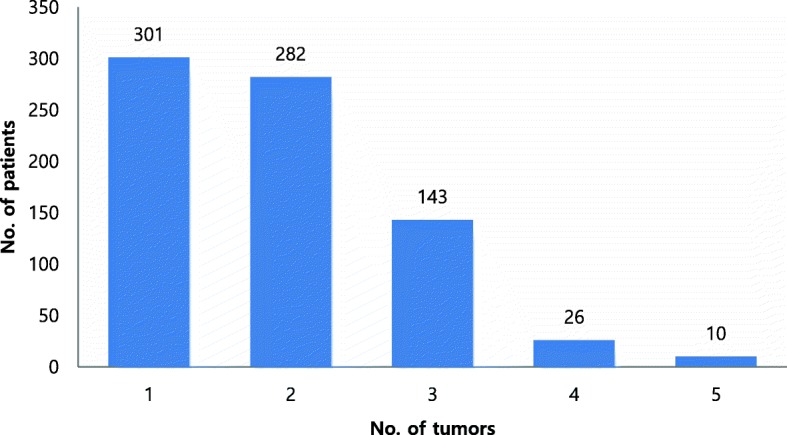
Distribution of 762 patients according to the number of tumors within the prostate gland

Among the 762 whole-mount specimens, 1448 tumor foci ranging in volume from 0.01 to 57.41 mL were identified. Table 2 shows the characteristics of the unifocal, primary, and secondary tumor foci. In multifocal disease, among the 686 secondary tumors, 250 (36.4%) had a GS ≥7 and 122 (17.8%) had a volume ≥ 0.5 mL. There were 166 (24.2%) cases with small tumor volume (< 0.5 mL) and Gleason pattern 4. Five secondary tumors were found to extend extra-capsularly, whereas the primary tumors were organ-confined. No secondary tumors were found to have invaded the seminal vesicles. Figure 1 shows the distribution of the number of tumors per prostate. A total of 583 (76.5%) patients had one or two tumors within the prostate gland.

**Table 2:** Histological characteristics of individual tumor foci from 762 radical prostatectomy specimens

Tumor type	Total	GS ≥7		GS ≤6		Volume ≥ 0.5 mL		ECE		SVI	
		*N*	%	*N*	%	*N*	%	*N*	%	*N*	%
Unifocal	301	269	89.4	32	10.6	254	84.4	59	19.6	46	15.2
Multifocal											
Primary tumors	461	384	83.3	77	16.7	371	80.5	69	14.9	23	4.9
Secondary tumors	686	250 (166)^a^	36.4 (24.2)^a^	436	63.6	122	17.8	5	0.7	0	0
Total	1448	903		545		747		133		69	

*ECE* = extracapsular extension, *GS* = Gleason score, *SVI* = seminal vesicle invasion

^a^Tumor with GS ≥7 and volume < 0.5 mL

The primary tumor was defined as the largest tumor as measured by volume, without considering its Gleason score. Secondary tumors were defined as all tumors that were smaller than the primary tumor

Of the 435 cases of bilateral multifocal disease, the secondary tumors on the contralateral lobes were significant by volume (≥0.5 mL) in 91 (20.9%) cases and by grade (GS ≥7) in 179 (41.1%) cases. There were 285 (65.5%) specimens with at least two tumor foci within the prostate gland with a different GS, and 29 (6.7%) specimens in which the secondary tumors on the contralateral lobe had a GS higher than the primary tumor score (Table 3). There were 102 (23.4%) tumors with a small tumor volume (< 0.5 mL) and Gleason pattern 4 on the lobe contralateral to the primary tumor.

**Table 3:** Cross-tabulation of histology (Gleason score) of primary and secondary tumors on the contralateral lobe to the primary tumor among patients with bilateral multifocal tumors (*n* = 435)

Primary tumor	Secondary tumor on the lobe contralateral to the primary tumor, *n* (%)				
	GS 3 + 3	GS 3 + 4	GS 4 + 3	GS ≥8	Total
GS 3 + 3	48 (11)	15 (3.4)	1 (0.2)	1 (0.2)	65
GS 3 + 4	149 (34.2)	92 (21.1)	8 (1.8)	3 (0.7)	252
GS 4 + 3	42 (9.6)	26 (6.0)	4 (0.9)	1 (0.2)	73
GS ≥8	21 (4.8)	14 (3.2)	7 (1.6)	3 (0.7)	45
Total	256	150	21	8	435

*GS* = Gleason score

### Comparison between unifocal and multifocal tumors

Table 4 compares patient and tumor characteristics in the unifocal and multifocal disease groups. Patients with unifocal tumors had significantly higher PSA levels (*p* = 0.023), larger tumor volumes (*p* < 0.001), and higher pathologic stages (*p* < 0.001) than those with multifocal tumors.

**Table 4:** Comparison between unifocal and multifocal tumors (*n* = 762)

	Unifocal	Multifocal	*P* value
N	301	461	
Age, years			
Mean (SD)	64.96 (7.07)	65.96 (6.50)	0.050
Median (range)	65 (43–80)	67 (45–81)	
PSA, ng/mL			
Mean (SD)	6.25 (3.25)	5.72 (2.94)	0.023
Median (range)	5.41 (0.37–14.78)	4.89 (0.34–14.95)	
Tumor volume, mL			
Mean (SD)	4.12 (5.47)	2.67 (2.88)	< 0.001
Median (range)	2.34 (0.01–57.41)	1.72 (0.01–23.13)	
Gleason Score ≥ 7	269 (89.4%)	407 (88.3%)	0.644
Stage ≥ T3a	106 (35.2%)	97 (21.0%)	< 0.001

*SD* = standard deviation

### Application of hemiablation in bilateral multifocal tumors

Of the 435 patients with bilateral multifocal tumors, 273 had organ-confined and GS ≤7 (3 + 4) cancer. If focal therapy (hemiablation) is performed with the aim of ablating the lobe where the tumor with the highest GS and largest volume is located, untreated lobes may continue to harbor small, clinically significant tumor (presence of Gleason pattern 4 and a volume < 0.5 mL), 22.3% (61/273) of which may not be detected by prostate biopsies and MRI findings before hemiablation.

## Discussion

Focal therapy provides an opportunity for effective treatment of prostate cancer with minimal morbidity by treating only the area of cancer or the index lesions of multifocal tumors while surveillance of the remaining clinically insignificant tumors is carried out. Although focal therapy is mainly performed in low-risk cases with small tumors that enable active surveillance, in the case of intermediate-risk cancers requiring active treatment, focal therapy has the advantage of providing comparable oncologic outcomes and low toxicity compared with treatment of the whole gland.

To the best of our knowledge, the present study is the first to address the issue of tumor focality, laterality, volume, and GS in a Korean setting for the purpose of considering focal therapy (hemiablation). In other studies, secondary tumors are characterized by small volumes and well-differentiated diseases. Bott et al. [21] reported tumor characteristics at RP for 77 men at low to intermediate risk for prostate cancer. A total of 11.7% (9/77) had unilateral cancer and 41/77 had multifocal cancer, with 33 of the multifocal patients having an index lesion in one lobe and clinically insignificant secondary tumors in the contralateral lobe. Among eight with clinically significant secondary tumors, six were clinically significant by GS (four contralateral, two ipsilateral) and two were significant by volume (one contralateral, one ipsilateral). Karavitakis et al. [22] reported a histopathological analysis of index and non-index lesions from 100 consecutive RP specimens. Among the 170 secondary lesions, 1 (0.6%) had a GS ≥7 and 22 (12.9%) were ≥ 0.5 mL. In both studies, no patient in had a secondary tumor with had a GS higher than the score of the index tumor of the specimen. However, our data showed several clinically significant secondary tumors (17.8% with tumor volume ≥ 0.5 mL and 36.4% with GS ≥7). The greatest difference compared with previous studies is that 29 (6.6%) of our cases had a secondary tumor with a GS higher than that of the primary tumor. Also, only three patients had a low-risk primary tumor (GS ≤6 and tumor volume < 0.5 mL) and secondary tumors with Gleason pattern 4. The present study highlighted that in Korean men with prostate cancer, secondary tumors frequently have a higher GS than reported by other studies.

Although the lack of prostate biopsy and mpMRI data in this study limits evaluation of patient selection for focal therapy, our study provides additional and important information on tumors in the lobe contralateral to the primary tumor. Among 14 cases of unilateral disease examined in a previous analysis of 100 whole-mount RP specimens [22], 10 patients were suitable for hemiablation; organ-confined, and GS ≤7 tumor. Among the 86 patients with bilateral disease, 35 with the index lesion confined to one lobe had secondary lesions in the contralateral lobe, with a tumor volume < 0.5 mL and GS ≤6. There were 45 patients in this series who could be considered suitable for hemiablation. However, in our study, if hemiablation can be delivered with the aim of ablating the lobe where the highest GS and larger tumor is located, and untreated lobes harboring clinically insignificant tumor undergo surveillance, 28% might have been appropriate for such a strategy. A possible explanation is that many clinically significant secondary tumors remain in the contralateral lobe. Because the proportion of unilateral disease is similar to that of Karavitakis [22] (11.5% vs. 10%).

Our study data found 41.1% of cases with a GS ≥7 and 20.9% with tumor volume ≥ 0.5 mL on the lobe contralateral to the primary tumor. More importantly, 23.4% of the cases involved small tumor volume (< 0.5 mL) and Gleason pattern 4 on the contralateral lobe, likely because mpMRI is less diagnostic for small lesions < 0.5 mL. Because Korean men have more significant small, secondary tumors in the contralateral lobe, accurate prostate biopsies are particularly important to establish focal therapy strategies. Sextant random biopsies are insufficient for accurate determination of tumor location within a prostate. Instead, perineal template-guided saturation biopsy has recently been preferred to aid with patient selection [23–25], and mpMRI has been used to select patients for clinical trials [26–28].

This study has several limitations. First, it was conducted retrospectively at a single institution, raising the possibility of selection bias. Second, it focused on histopathological analysis of RP specimens and did not include preoperative data (e.g., clinical staging and biopsy findings). Although we considered only patients with a PSA < 15 ng/mL in an effort to exclude cases of advanced prostate cancer, some patients who were not considered suitable candidates for focal therapy even before RP could have been included in the patient population. Future research should analyze RP specimens by applying additional patient selection criteria such as a preoperative PSA level < 15 ng/mL, clinical stage <T2c, GS ≤3 + 4, and a Prostate Imaging Reporting and Data System score < 4 on the contralateral lobe based on mpMRI. Our study has the additional limitation that clinical outcomes were not included in the study protocol, therefore no definitive conclusions can be made regarding the effects of tumor focality and laterality on disease progression. However, a group from Duke University demonstrated that prostate cancer laterality does not affect disease progression [29].

## Conclusions

Bilateral and multifocal tumors are common features on RP specimens. Total tumor volume, GS, extracapsular extension, and seminal vesicle invasion are almost always determined by the index tumor. However, secondary tumors in Korean men frequently present with clinically significant prostate cancer. Furthermore, we observed many cases in which secondary tumors on the lobe contralateral to the primary tumor had a higher GS and small tumor volume. Additional studies are required to evaluate the accuracy of combined mpMRI and transperineal template-guided saturation biopsies in patient selection for application of hemi-ablative focal therapy.

## Abbreviations

GS: Gleason score
PSA: Prostate-specific antigen
RP: Radical prostatectomy
